# Supplementary material for: Orf Virus-Based Vaccine Vector D1701-V Induces Strong CD8+ T Cell Response against the Transgene but Not against ORFV-Derived Epitopes
Source: Vaccines (Basel). 2020 Jun 10;8(2):295. doi: 10.3390/vaccines8020295 (PMC7349966; doi:10.3390/vaccines8020295)
Supplement: Supplementary file 1 [file vaccines-08-00295-s001.pdf]

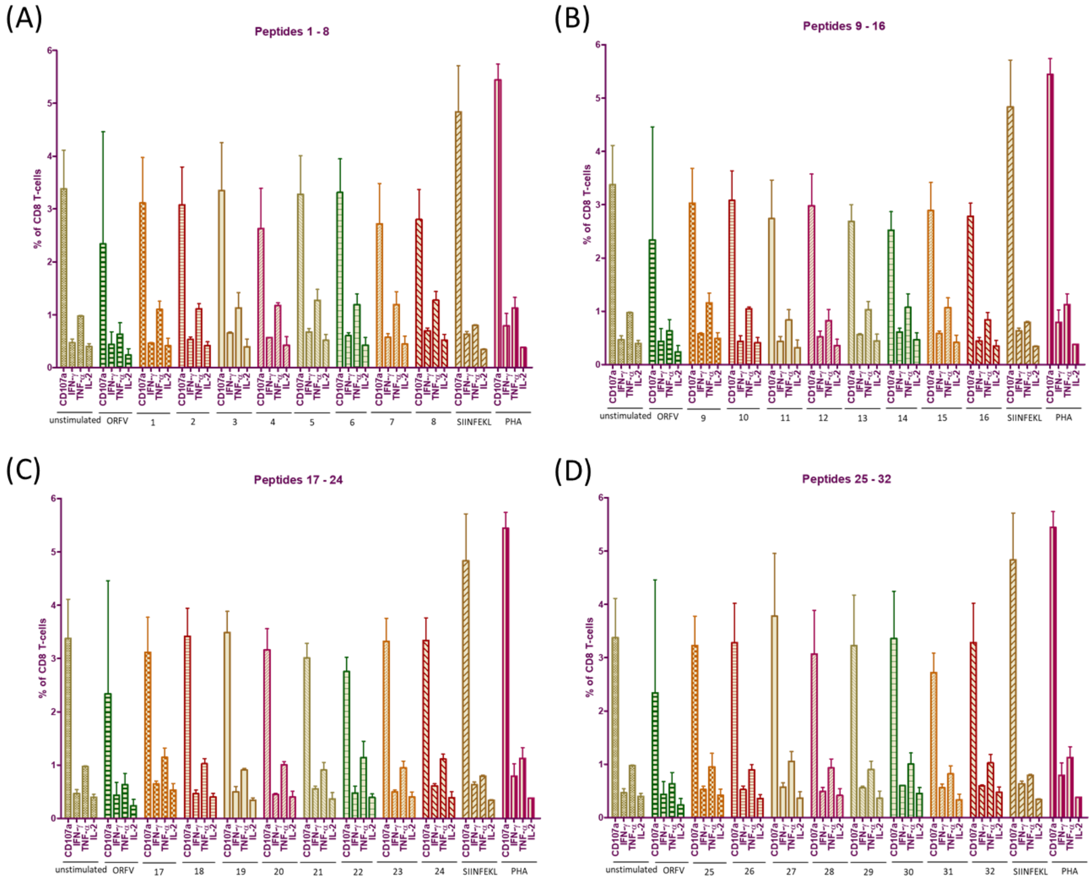

Figure S1. CD8+ T cell responses within 14 days re-stimulated splenocytes from ORFV immunized mice. H-2K^b C57BL/6 mice (n = 6) were immunized twice with V12-Ova-D12-GFP or control ORFV by i.m. route. One week after the second administration isolated pooled splenocytes were re-stimulated during 14 days with the individual ORFV-derived peptides as described in Materials and Methods. Specific cytotoxic T lymphocyte responses were evaluated using intracellular cytokine staining. Percentages of CD107a, TNF-α, IFN-γ and IL-2 expressing CD8+ T cells after stimulation with synthetic peptides (**A**) 1–8, (**B**) 9–16, (**C**) 17–24 and (**D**) 25–32, control ORFV or Ova$_{257\text{-}264}$ SIINFEKL peptide. Data are shown as means ± SEM of three independent technical replicates. PHA, Phytohemagglutinin; ns, not significant; Ova, ovalbumin; TNF-α, tumor necrosis factor alpha; IFN-γ, interferon-gamma; IL-2, interleukin-2.
